# Detection of Tissue Factor Antigen and Coagulation Activity in Coronary Artery Thrombi Isolated from Patients with ST-Segment Elevation Acute Myocardial Infarction

**DOI:** 10.1371/journal.pone.0081501

**Published:** 2013-12-11

**Authors:** Tullio Palmerini, Luciana Tomasi, Chiara Barozzi, Diego Della Riva, Andrea Mariani, Nevio Taglieri, Ornella Leone, Claudio Ceccarelli, Stefano De Servi, Angelo Branzi, Philippe Genereux, Gregg W. Stone, Jasimuddin Ahamed

**Affiliations:** 1 Dipartimento Cardiovascolare, Policlinico S. Orsola, Bologna, Italy; 2 Istituto di Patolgia, Policlinico S. Orsola, Bologna, Italy; 3 Dipartimento di Radiologia e Scienza Istopatologiche, Policlinico S. Orsola, Bologna, Italy; 4 Dipartimento di Malattie Cardiovascolari, Ospedale Civile, Legnano, Italy; 5 Columbia University Medical Center and the Cardiovascular Research Foundation, New York, New York, United States of America; 6 Laboratory of Blood and Vascular Biology, The Rockefeller University, New York, New York, United States of America; University Heart Center Freiburg, Germany

## Abstract

**Introduction:**

Although ruptured atherosclerotic plaques have been extensively analyzed, the composition of thrombi causing arterial occlusion in patients with ST-segment elevation acute myocardial infarction has been less thoroughly investigated. We sought to investigate whether coagulant active tissue factor can be retrieved in thrombi of patients with STEMI undergoing primary percutaneous coronary intervention.

**Methods:**

Nineteen patients with ST-segment elevation acute myocardial infarction referred for primary percutaneous coronary intervention were enrolled in this study. Coronary thrombi aspirated from coronary arteries were routinely processed for paraffin embedding and histological evaluation (4 patients) or immediately snap frozen for evaluation of tissue factor activity using a modified aPTT test (15 patients). Immunoprecipitation followed by immunoblotting was also performed in 12 patients.

**Results:**

Thrombi aspirated from coronary arteries showed large and irregular areas of tissue factor staining within platelet aggregates, and in close contact with inflammatory cells. Some platelet aggregates stained positive for tissue factor, whereas others did not. Monocytes consistently stained strongly for tissue factor, neutrophils had a more variable and irregular tissue factor staining, and red blood cells did not demonstrate staining for tissue factor. Median clotting time of plasma samples containing homogenized thrombi incubated with a monoclonal antibody that specifically inhibits tissue factor-mediated coagulation activity (mAb 5G9) were significantly longer than their respective controls (88.9 seconds versus 76.5 seconds, respectively; p<0.001). Tissue factor was also identified by immunoprecipitation in 10 patients, with significant variability among band intensities.

**Conclusions:**

Active tissue factor is present in coronary artery thrombi of patients with ST-segment elevation acute myocardial infarction, suggesting that it contributes to activate the coagulation cascade ensuing in coronary thrombosis.

## Introduction

ST-segment elevation myocardial infarction (STEMI) is one of the leading causes of mortality and morbidity in the western world [1]. There is increasing evidence that STEMI is in most cases precipitated by rupture of thin cap fibroatheromas, and that the exposure of procoagulant material contained in the plaque to circulating blood initiates platelet aggregation, thrombin generation and fibrin deposition ultimately leading to coronary thrombosis [2]. However, while ruptured atherosclerotic plaques have been extensively analyzed [3], [4], [5], the composition of thrombi causing arterial occlusion has been less thoroughly investigated.

Tissue factor (TF) is a glycoprotein that serves as the initiator of the coagulation cascade. TF binds the serine protease factor VII to activate factor X, leading to thrombin generation and thrombus formation [6]. Alternatively, TF can bind protease-activated receptors (PARs) to signal inflammation and tumorigenesis [7], [8]. TF is normally localized in extracellular spaces such as in adventitial cells of blood vessels, epidermal cells of skin, fibrous capsule, and stromal cells, thus providing a continuous hemostatic barrier ready to initiate the coagulation cascade upon vessel wall integrity disruption [9]. TF is therefore considered a major regulator of coagulation, haemostasis, and thrombosis [10]. TF antigen has also been identified in atherosclerotic plaques [4], [11], [12], but less is known about its presence and activity in thrombosis during STEMI. In this study we analyzed the presence of TF antigen and its coagulation activity in thrombi harvested from coronary arteries of patients with STEMI referred for primary percutaneous coronary intervention (PCI).

## Methods

### Ethic Statement

The present clinical investigation was conducted according to the principles expressed in the Declaration of Helsinki. The study was approved by the ethics committee of Policlinico S.Orsola of Bologna, and all patients provided written informed consent to participate in the study.

### Design and Patient Selection

Patients were eligible for participation if they were aged 18 years or older, presented within 12 hours after the onset of symptoms, had ST-segment elevation of 1 mm or more in 2 or more contiguous leads, had a total coronary occlusion (TIMI flow = 0), and were candidates for thrombus aspiration with a manual thrombectomy device. Exclusion criteria were the presence of cardiogenic shock or inability to obtain written informed consent. The study consisted of 2 groups: in the first group (4 patients), thrombi harvested from coronary arteries were paraffin embedded and the presence of TF antigen was evaluated by immunohistochemistry. In the second group (15 patients), thrombi were snap frozen in liquid nitrogen and subsequently analyzed with clotting assays to assess the presence of TF activity.

### Reagents

Pooled normal human plasma was prepared from blood collected into 4 ml vacuum tubes containing sodium citrate (3.2%) from 15 healthy volunteers. Blood was immediately centrifuged at 3,000 g×10 minutes at room temperature. Plasma samples were then pooled, aliquoted, and frozen at −20°C. Tris-buffer saline (TBS) was prepared using 50 mM Tris-HCl, 150 mM NaCl, pH 7.4. Antibodies were described previously [8], [13], [14]. Monoclonal antibody (mAb) 5G9 inhibits coagulation activity of TF [8], [15]; mAb 10H10 recognizes the noncoagulant, cryptic conformation of TF, inhibiting TF-VIIa mediated signaling via PAR2 [16]. Although mAb 5G9 reacts with both coagulant and cryptic forms of TF on cells, it does not inhibit TF-VIIa signalling. In sharp contrast, mAb 10H10 shows poor reactivity towards coagulant active TF, but efficiently blocks TF-VIIa signaling. TF mAb 9C3 and affinity purified goat anti-TF antibodies were described previously by Ahamed et al [16]. The anti-TF mAb CD 142 is specific for aminoacids 1–25 within the extracellular domain (American Diagnostica, Stamford, CT). Chromo Pure mouse IgG was from Jackson Immunoresearch Labs Inc. (West Grove, PA). Commercial thromboplastin (RecombiPlasTin 2G, Instrumentation Laboratory, Bedford, MA) was used to determine prothrombin times in control experiments.

### PCI Procedure

PCI was performed according to standard technique. After wiring the coronary obstruction, coronary thrombi were aspirated using a manual thrombectomy device (ELIMINATE, Terumo Clinical Supply, Kakamigahara, Japan). The aspiration catheter was passed through the coronary obstruction 3 times, starting the aspiration right before the obstruction. All patients were given aspirin 250 mg intravenously, and heparin 70 U/kg before thrombus aspiration. Use of glycoprotein IIb/IIIa inhibitors as well as stent choice was left at physician’s discretion. A loading dose of clopidogrel 600 mg was administered at the end of the procedure in all patients.

### Coronary Thrombi

Thrombi aspirated from coronary arteries were harvested using a filter basket supplied by the manufacturer of the aspiration device, and then either snap frozen in liquid nitrogen and stored at −80° until analysis, or immediately fixed in formalin. The day of analysis, snap frozen thrombi were homogenized using a tissue homogenizer (Wheaton Industries Inc, Millville, NJ), and resuspended in TBS. The protein concentrations were adjusted to 10 mg/ml. Then, 20 µl was used for the clotting assay, and the remainder volume lysed for immunoprecipitation followed by immunoblotting. Formalin-fixed thrombi were processed for histologic analysis.

### Histology and Immunohistochemistry

Thrombus aspirate specimens were fixed in 10% paraformaldehyde, and paraffin embedded. From each paraffin block, sections (2 µm) were cut and stained with haematoxylin-eosin and Azan-Mallory trichrome. Immunohistochemistry was performed using 2 different antibodies: CD142 (dilution 1∶250) and 10H10 (dilution 1∶100). For CD142 immunostaining, sections were treated with citrate buffer, pH 6.0 at 98°C for 40 minutes. Anti-CD142 immunostaining was done with DakoCytomation autostainer using Dako Advance visualization System. For 10H10 staining, sections were treated with Tris-EDTA buffer, pH 9.0 at 98°C for 20 minutes. Anti-10H10 immunoperoxidase staining was manually performed with overnight incubation at room temperature using Novolink visualization system (Novocastra Laboratories, Newcastle UK). A negative control was performed using an isotype-matched irrelevant primary antibody (mouse IgG anti-epithelial membrane antigen; Ventana Medical Systems, Inc, Tucson, AZ).

### Clotting Assay

TF activity of homogenized thrombi was analyzed using a modified activated partial thromboplastin time (aPTT) test in an automated optical instrument (ACL ADVANCE, Instrumentation Laboratory, Milan, Italy). Pooled normal human plasma (60 µl) was incubated with homogenized thrombi solution (10 µl) with or without mAb 5G9 (300 µg/ml final concentration) for 10 minutes at room temperature. Then, 100 µl of liquid rabbit cephalin (ACTIN, Siemens Healthcare diagnostic, Marburg, Germany), diluted 1∶1 with 0.025 mol/L CaCl_2_ was added and the time to coagulation measured. Control samples included plasma with buffer, 5G9, 10H10 or IgG. As a quality control we also assessed the effect of mAb 5G9 on exogenously added TF- mediated clotting activity using standard prothrombin times.

### Immunoprecipitation and Immunoblotting of TF

Proteins from coronary artery thrombi were extracted after homogenization in buffer containing 50 mM *n*-octyl *β*-D-glucopyranoside for 1 hour on ice. TF was immunoprecipitated from the lysed thrombi using mAb 9C3 coupled to magnetic Dynabeds (Invitrogen). Immunoblotting was performed with affinity purified goat anti-TF antibody. Normalization of western blot analysis was performed by loading the same amount of total proteins (20 µg). Blots were digitized for densitometry by using ImageJ program (NIH).

### Statistical Analysis

Data are presented as mean ± SD or median and interquartile range as appropriate. Normality was checked with the Smirnov-Kolmogorov test. Continuous data were compared by pairwise Student t test or Wilcoxon Signed Rank test, as appropriate. Correlations were evaluated with the Spearman test. Statistical analyses were performed using Stata/SE 11.2 (StataCorp LP, College Station TX). P values<0.05 were considered statistically significant.

## Results

### Patients

Of the 24 patients considered eligible for the study in the period between February 2012 and August 2012, 5 were excluded because aspirates did not contain particulate thrombi sufficient for analyses, and 19 were finally included in the study. The clinical characteristics of patients enrolled in this study are reported in Table 1. The median age was 67 years and 12 of the 19 patients were male. Median time of ischemia was 150 minutes with a range of 30 to 720 minutes. No major complications occurred during the procedure and all patients were discharged alive.

**Table 1:** Baseline clinical characteristics of patients recruited in the study.

Age, years		67 (58–75)
Male		63.2% (12/19)
History of CAD		26.3% (5/19)
Hypertension		57.9% (11/19)
Diabetes mellitus		21.1% (4/19)
Hypercholesterolemia		46.6% (7/19)
Smoking		63.2% (12/19)
Prior MI		0.0% (0/19)
Prior PCI		0.0% (0/19)
Peripheral vascular disease		0.0% (0/19)
Renal dysfunction		0.0% (0/19)
Left ventricular ejection fraction		45% (40%–55%)
Killip class	I	94.7% (18/19)
	II	5.3% (1/19)
	III	0.0% (0/19)
	IV	0.0% (0/19)
Access site	Femoral	57.9% (11/19)
	Radial	42.1% (8/19)
Total ischemic time (mins)*		150 (108.5–450.5)
MI site	Anterior	68.4% (13/19)
	Inferior	31.6% (6/19)
Culprit artery	LAD	68.4% (13/19)
	RCA	21.1% (4/19)
	Cx	10.5% (2/19)
Number of diseased vessel		1.4±0.6
Number of treated vessels		1.1±0.5
Pre-procedural TIMI flow = 0		100% (19/19)
Post-procedural TIMI flow	0/I	0.0% (0/19)
	II	15.8% (3/19)
	III	84.2% (16/19)
Treatment with bare metal stents		94.7% (18/19)
Treatment with drug-eluting stents		5.3% (1/16)
Therapy at hospital admission	Aspirin	10.5% (2/19)
	Beta blockers	10.5% (2/19)
	ACE inhibitors	36.8% (7/19)
	ARBs	15.8% (3/19)
	Calcium antagonist	15.8% (3/19)
	Diuretics	21.1% (4/19)
	Statins	5.3% (1/19)
	Proton pump inhibitors	5.3% (1/19)
Intraprocedural therapy	Intravenous aspirin	100% (19/19)
	UFH (70 UI/Kg)	100% (19/19)
	Glycoprotein IIb/IIIa inhibitor	100% (19/19)

Continuous data are presented as median (interquartile range). MI denotes myocardial infarction; CAD denotes coronary artery disease; PCI denotes percutaneous coronary intervention; LAD denotes left anterior descending coronary artery; RCA denotes right coronary artery; Cx denotes circumflex artery; UFH denotes unfractionated heparin.

* total ischemic time denotes time from symptom onset to balloon dilatation in minutes.

### Histological Evaluation

**Figure 1:**
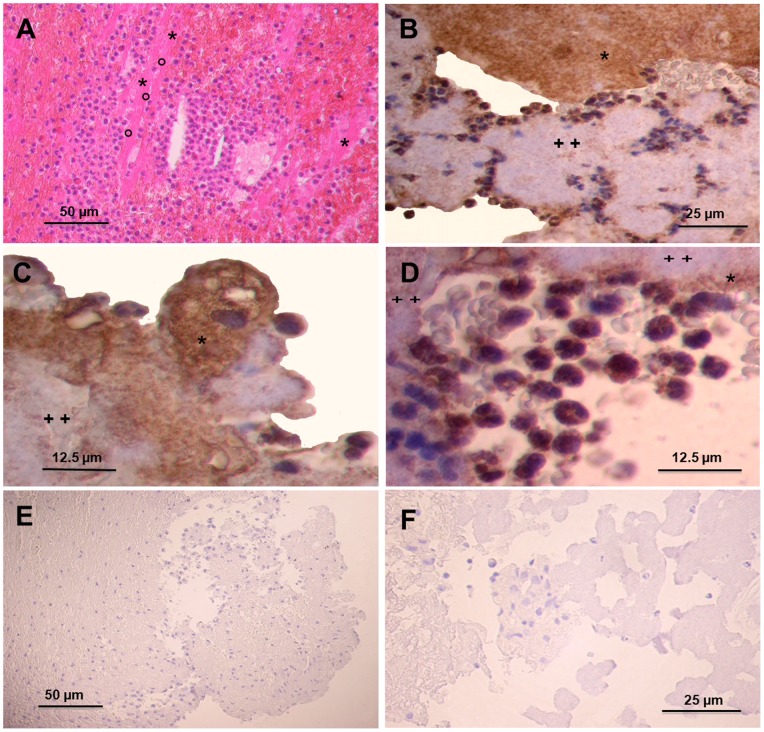
Histological assessment of thrombi aspirated from coronary arteries of patients with ST-segment elevation acute myocardial infarction. (A) Hematoxylin and eosin staining shows platelet aggregates (*) intermingled in a fibrin network (°) with red and white blood cells. Original magnification 100x. (B) and (C) Immunohistochemistry of coronary thrombi prepared with anti-TF CD142 antibody showing large and irregular areas of tissue factor staining (brown colour) within platelet aggregates and white blood cells. Some portion of the platelet aggregates stained intensely (*), whereas other lightly (++) with anti-TF CD142 antibody. B: original magnification 200x; C: original magnification 400x. (D) Detail of a coronary thrombus showing tissue factor staining in close contact with platelet aggregates (*). Some areas of these platelet aggregates do not display tissue factor staining (++). Monocyte are strongly positive for tissue factor, granulocytes display a weak and irregular staining, and red cells are negative. Original magnification 400x. (E) Negative control for TF performed with immunoperoxidase staining on sections not incubated with the primary antibody against TF. Magnification 100x. (F) Negative control using an isotype-matched irrelevant primary antibody (mouse IgG anti-epithelial membrane antigen).

**Figure 2:**
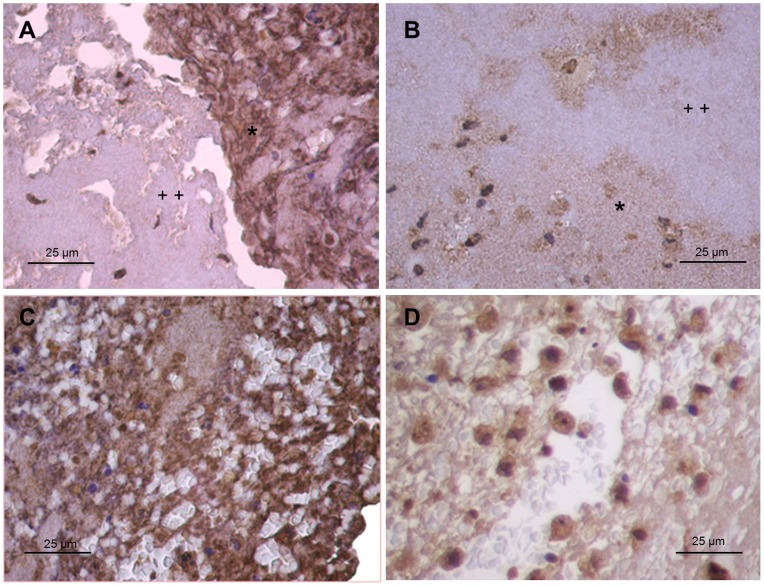
Immunohistochemistry of coronary thrombi. (A,B and C) Immunohistochemistry prepared with 10H10 showing large and irregular areas of tissue factor staining (in brown) within platelet aggregates and inflammatory cells. Some platelet aggregates stained positive for tissue factor (*), whereas other did not (++). Original magnification is ×200. (D) Detail of a coronary thrombus prepared with 10H10. Monocytes are strongly positive for tissue factor, granulocytes display a weak and irregular staining, and red cells are negative. Original magnification is ×400.

Histological evaluation of thrombi aspirated from coronary arteries of 4 patients with STEMI showed irregular areas of erythrocytes trapped in a fibrin network with large and irregular areas of platelet aggregates and clusters of granulocytes and monocytes (Figure 1A–D). Samples stained with CD142 showed large and irregular areas of TF within platelet aggregates, and in close contact with inflammatory cells (Figure 1B and C). Of note, some platelet aggregates stained positive for TF, whereas others did not (Figure 1B). Monocytes stained intensely, neutrophils gave a more variable staining, and red blood cells were negative (Figure 1D). The negative control for TF staining is provided in Figure 1E. TF staining with mAb 10H10, which has higher affinity for the signalling form of TF, showed similar staining pattern as CD142 (Figure 2). In particular, some platelet aggregates stained positive for 10H10, whereas other did not (Figure 2A and B). Monocytes stained strongly, neutrophils weakly, and red blood cells negatively (figure 2C).

### TF Activity in Thrombi of Patients with STEMI

**Figure 3:**
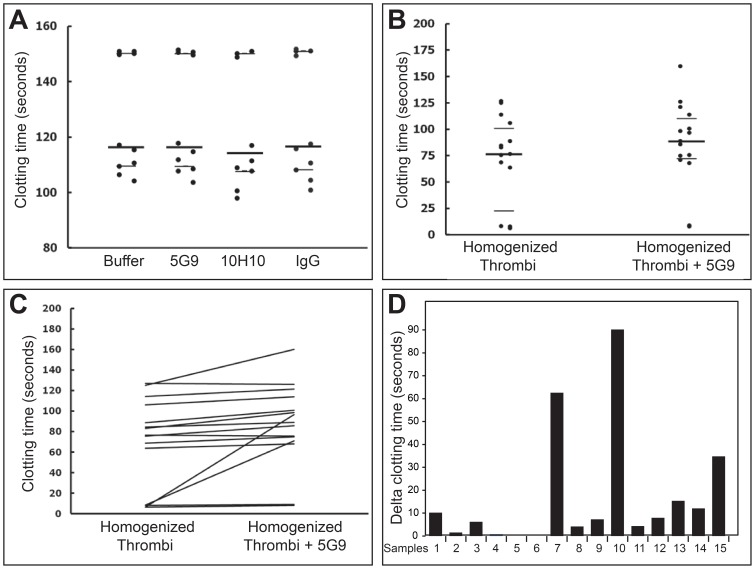
Clotting times measured with the modified aPTT test. (A) Clotting times of plasma samples incubated with buffer, 5G9, 10H10 or IgG. (B) Clotting times of plasma samples containing homogenized thrombi incubated with or without 5G9. (C) Pairwise comparison of clotting times of plasma samples containing homogenized thrombi incubated with or without 5G9. (D) Differences in clotting times between plasma samples containing homogeneized thrombi incubated with or without 5G9.

In the quality control experiments, mAb 5G9 inhibited clotting activity of exogenous TF in a dose dependent manner (data not shown). In sharp contrast (Figure 3A), no significant difference was apparent in clotting times of plasma samples not containing homogeneized thrombi and incubated with either 5G9 (median 116.3 seconds, IQ range 108.6–150.6 seconds), TBS (median 116.3 seconds, IQ range 109.5–150.2 seconds), 10H10 (median 114.2 seconds, IQ range 107.8–150.2 seconds), or an isotype-matched IgG (median 116.7 seconds, IQ range 108.2–151.1 seconds). All these clotting times were significantly longer when compared to clotting times of plasma samples containing homogenized thrombi not incubated with 5G9 (p<0.001).

Clotting times of plasma samples containing homogeneized thrombi were non normally distributed, varying from 6.4 seconds to 126.9 seconds in plasma samples containing homogenized thrombi not incubated with mAb 5G9, and from 8.0 to 160.0 seconds in those incubated with mAb 5G9. Clotting times of plasma samples containing homogenized thrombi incubated with 5G9 were significantly longer than samples without mAb 5G9 (Figure 3B). In particular, median clotting time was 88.9 seconds (IQ range 71.9–110.6 seconds) for homogenized thrombi incubated with mAb 5G9 versus 76.5 seconds (IQ range 22.3–101.7 seconds) for homogenized thrombi not incubated with mAb 5G9 (p<0.001). Incubation of thrombi homogenates with mAb 5G9 increased clotting times in 13 out of 15 patients (Table 2 and Figure 3C). Differences in clotting times (delta clotting time) between homogenized thrombi incubated with or without the antibody varied significantly from sample to sample, ranging from 0.9 seconds to 90.1 seconds (Figure 3D).

**Table 2:** Clotting times and densitometric quantification of tissue factor in coronary thrombi of patients included in the study.

	Clotting times of homogenized thrombi	Clotting times of homogenized thrombi +5G9	Delta (seconds)	Densitometric quantification of TF by immunoprecipitation (AU)
Patient 1	75.6	85.8	10.2	NA
Patient 2	6.4	8.0	1.6	3,764
Patient 3	68.9	75.1	6.2	NA
Patient 4	8.0	8.9	0.9	1,943
Patient 5	126.9	126.1	−0.8	1,023
Patient 6	76.5	75.6	−0.9	4,660
Patient 7	8.4	70.9	62.6	9,200
Patient 8	63.9	68.1	4.2	219
Patient 9	114.1	121.4	7.3	10,219
Patient 10	6.6	96.7	90.1	36,457
Patient 11	84.5	88.9	4.4	4,710
Patient 12	106.0	113.9	7.9	NA
Patient 13	83.0	98.4	15.4	5,640
Patient 14	88.7	100.8	12.1	9,091
Patient 15	125.2	160.0	34.8	155

Clotting times are provided in seconds; AU denotes arbitrary unit; NA denotes not available.

### Western Blot

**Figure 4:**
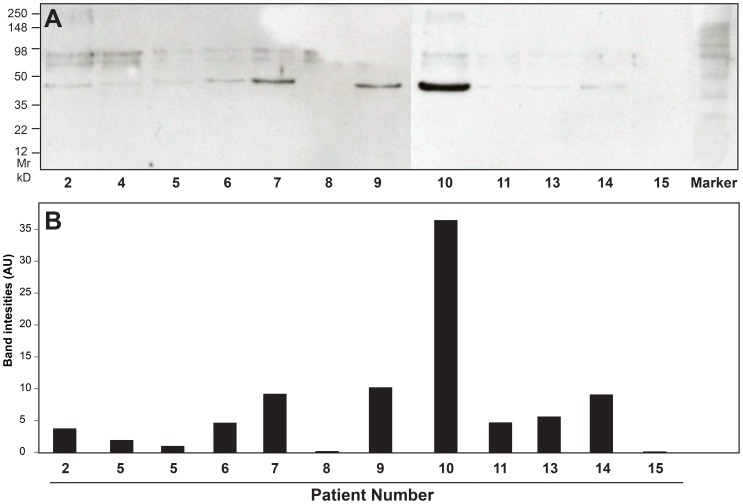
Immunoblotting analysis. (A) Immunoblotting of thrombi harvested from 12 patients showing the presence of various amount of tissue factor antigen (47 kD). (B) Band intensity of the immunoblot expressed in arbitrary unit (AU).

TF was detected by immunoblotting in 10 out of the 12 patient samples tested, but the staining intensity varied (Figure 4). Eight out of 10 samples with detectable TF antigen by immunoblotting also showed TF coagulant activity. There was no significant correlation, however, between TF antigen levels measured by immunoblotting and TF clotting activity measured by aPTT assay, although a trend was apparent (correlation coefficient = 0.52; p = 0.08).

## Discussion

Our data provide direct evidence that both TF antigen and TF coagulation activity are present in thrombi isolated from patients with STEMI. We developed a new method to assess TF activity in thrombi harvested from patients with STEMI. It uses the same principle as a conventional aPTT assay, but adds a mAb specific for TF clotting activity to assess specificity. Control experiments demonstrated that mAb 5G9 does not affect the clotting times of plasma samples not containing homogenized thrombi, but inhibits exogenously added TF.

Clinical and experimental studies have demonstrated the presence of TF antigen in animal models of stent thrombosis [17], in human arterial specimens [3], [5], and in coronary atherectomy specimens retrieved from patients with unstable angina or myocardial infarction [4], [11], [12]. Some of these studies have also suggested that TF contained in atherosclerotic plaque is potentially active [3], [4], [12]. Although these prior observations suggest that TF may be an important determinant of plaque thrombogenicity, no study has ever provided direct evidence that TF contributes to thrombus formation and propagation in STEMI. Our study for the first time provides direct evidence that coagulation active TF accumulates in the coronary thrombi isolated from patients with STEMI, thus triggering the coagulation cascade that leads to coronary thrombosis. Two prior studies have shown the presence of TF antigen within thrombi harvested from coronary arteries of patients with STEMI, but none of them demonstrated TF activity, thus leaving undetermined whether TF may contribute to thrombus growth in this particular setting [18], [19]. Our findings may have important pathophysiological and clinical implications, suggesting that TF blockade may be an effective therapeutic strategy to prevent coronary thrombosis.

An important question raised by our study concerns the source of TF. TF is normally separated from the blood by the vascular endothelium [10]. However, this paradigm has been challenged by the demonstration that TF is also present in circulating blood [20], [21], [22]. Our study was not designed to address the source of TF found within coronary thrombi, and therefore no conclusion can be drawn from our findings. However, although we cannot exclude the vessel wall or the ruptured plaque as the source of TF, the distribution of TF within thrombi, in close contact with platelet aggregates or inflammatory cells suggests that at least in part, some of TF that is present in thrombus may be blood borne. Thus, cryptic circulating TF may be recruited and become biologically active at site of thrombus formation, thus contributing to its growth and propagation. This hypothesis is consistent with experimental studies that have demonstrated incorporation of active circulating TF in growing thrombi generated in animal models [23], or in ex-vivo perfusion systems [20], [21], [24]. Recent data have also suggested that monocytes and possible granulocytes are possible sources of circulating TF [25]. Leukocytes can transfer TF to platelets via CD15 receptors, forming highly procoagulant platelet aggregates containing leukocyte-derived TF [26]. In addition, Camera et al. have suggested that platelets themselves are able to express functionally active TF, making them capable of triggering the coagulation cascade [27], [28], [29]. Of note, in our study TF staining was noted in close contact and within platelet aggregates, suggesting that platelets play an important role in supporting TF-dependent coagulation.

TF activity within coronary thrombi varied greatly across samples, possibly reflecting variable distribution of TF within coronary thrombi, and aspiration of only part of the total volume of thrombi. Of note, in 2 cases we could not demonstrate the presence of TF by immunoprecipitation, even though TF could be measured by the clotting assay. Moreover, no significant correlation between TF antigen and TF activity was apparent, confirming our previous finding that very little TF is required for coagulation activity [16].

Intriguingly, both the procoagulant and the signalling conformation of TF were found within coronary thrombi, as demonstrated by 10H10 staining [16]. This finding may explain the absence of correlation between TF activity measured with clotting assays and TF antigen identified with immunoblotting. TF has in fact alternative conformations with distinct functional properties depending on the surface accessible, extracellular Cys^186^–Cys^209^ disulfide bond [16]. The non-coagulant cryptic conformation of TF mediates PAR2-dependent TF/VIIa signalling [30]. Of note, in our study TF staining within coronary thrombi using 10H10 displayed a similar pattern as with CD142 staining. Some experimental studies have suggested that TF-dependent PAR2-activation may increase infarct size in a mouse model of cardiac ischemia/reperfusion injury [31]. Based on our previous study demonstrating two antigenically distinct conformations of TF recognized by the two mAbs 5G9 and 10H10 [16], we propose that both pools of TF are present in the thrombi isolated from patient with STEMI. Further studies are warranted to investigate the role and the source of these two pools of TF.

This study has some limitations that should be acknowledged. Because thrombi were retrieved by an aspiration catheter, we could analyze only large aspirated pieces, and not the total volume of the occlusive thrombi. The study had several exclusion criteria and therefore whether our findings may be extrapolated to all STEMI patients is unknown. The absence of successful thromboaspiration in all STEMI patients may also have biased our results. We used TBS as control for 5G9 specificity. However, an isotype-matched control antibody would have been more appropriate. We did not investigates the source of TF and therefore further study is warranted to address this issue. All patients were treated with bare metal stents and clopidogrel, which do not represent any more the standard of care for patients with STEMI, meta-analysis and randomized trials suggesting that second generation drug-eluting stents [32], and new and more potent antiplatelet therapies [33], [34] might significantly improve the outcomes of this high risk cohort of patients.

In conclusion, our study clearly demonstrates the presence of active TF within coronary thrombi of patients with STEMI, suggesting that TF may contribute in triggering the coagulation cascade that ultimately leads to coronary thrombosis development.
